# Small-Scale Spatial Distribution of Mountain Pine Beetle Attacks by Parent and Brood Adults in Lodgepole Pine Forests in Northern Colorado

**DOI:** 10.3390/insects17060560

**Published:** 2026-05-29

**Authors:** José F. Negrón, Larry Scott Baggett

**Affiliations:** USDA Forest Service, Rocky Mountain Research Station, Fort Collins, CO 80526, USA

**Keywords:** *Dendroctonus ponderosae*, *Pinus contorta*, bark beetles, beetle attacks

## Abstract

The mountain pine beetle, *Dendroctonus ponderosae*, is a bark beetle that can cause extensive tree mortality of its hosts in western North America. Lodgepole pine, *Pinus contorta*, is one of its primary and most widely distributed hosts. The insect exhibits a one-year life cycle with a dispersal flight of emerging adults, referred to as brood adults, which attack new trees in the late spring–early summer. The larvae develop through the summer and overwinter. The following spring, development resumes, followed by pupation and emergence of a new generation of brood adults. Every year, a proportion of adults survive the winter, referred to as parent adults, re-emerge and attack new trees prior to brood adult emergence. These contribute little to population dynamics, but it is unknown whether these parent adults contribute to host finding by brood adults by initiating attacks that lead to clustering of attacks by brood adults. We tested this hypothesis by sequentially marking attacked trees from early spring to the fall in 2006, 2008, and 2015 in stands in northern Colorado. The sudden increase in the number of attacks indicates brood adult emergence. We tested for clustering of parent adult- and brood adult-attacked trees and of brood adult-attacked trees around parent adult-attacked trees using Ripley’s K-function. We found evidence of clustering of parent adult-attacked trees and brood adult-attacked trees, but there is no evidence of clustering of brood adult-attacked trees around parent adult-attacked trees.

## Simple Summary

The mountain pine beetle is a bark beetle that attacks various species of pine in western North America. Lodgepole pine is one of its primary and most widely distributed hosts. Low-level populations usually attack small numbers of stressed trees, but high-level widespread populations can cause extensive mortality. The insect exhibits a one-year life cycle, with adults emerging in large numbers during its primary dispersal flight and attacking new trees in late spring–early summer. Larvae develop through the summer, overwinter, and repeat the cycle the next year. A proportion of old adults survive the winter and attack new trees in the spring prior to the primary dispersal flight. Although they contribute little to population development, their role as potentially leading later-emerging beetles in host finding has not been thoroughly explored. In this study, we followed early- and late-emerging adults during the primary flight to examine clustering of parent adult-attacked trees, brood adult-attacked trees around parent adult-attacked trees, and brood adult-attacked trees. We found evidence of clustering of parent adult- and brood adult-attacked trees but no evidence of clustering of brood adult-attacked trees around parent adult-attacked trees.

## 1. Introduction

Dispersal of phytophagous insects into previously unexploited suitable habitats that provide new hosts is crucial to maintaining viable insect population levels. Naturally, without this capacity, a species would be on a trajectory toward extinction. For insects that utilize trees as habitat, such as bark beetles, leaving a no-longer-viable host in search of a new host is a precarious phase of their life cycle. At this time, they become prey to avian species and other organisms and may be exposed to unsuitable environmental conditions. In addition, flights from one location to another are physiologically demanding. The species needs to develop adaptive strategies that allow for successful dispersal and invasion of new hosts for reproduction and survival.

The mountain pine beetle (MPB), *Dendroctonus ponderosae* Hopkins (Curculionidae: Scolytinae), is a widely distributed bark beetle across coniferous forests of western North America that utilizes species of pines (*Pinus* spp.) as hosts [1]. Lodgepole pine (*Pinus contorta* Dougl. var. *latifolia* Engelm.) and ponderosa pine (*Pinus ponderosa* Lawson & C. Lawson) are the most abundant and geographically distributed hosts. Low-level populations are commonly found in stressed trees [2,3,4] such as those affected by diseases or lightning strikes. Episodic eruptive populations can cause extensive tree mortality across large areas. From the late 1990s through the mid-2010s, during an extensive transcontinental MPB outbreak, millions of acres were affected in British Columbia and the western United States, including Colorado [5,6,7,8].

MPB most commonly has one generation per year, although at high elevations and latitudes, a semi-voltine lifecycle can be observed. In contrast, the development of more than one generation in one year, although unusual, can occur under particularly warm conditions [9,10,11,12]. The one-year life cycle begins with a dispersal event when beetles emerge from trees infested the previous year. In Colorado, this dispersal flight occurs primarily in late July–early August, although variability occurs with geographical locations and elevation. The timing of the emergence period from year to year can vary by days [9,13]. Emerging insects colonize new trees through a highly complex host selection process that includes random landings on trees, gustatory and visual cues, attraction to host monoterpenes, and insect-produced pheromones [14,15,16]. Once a tree is determined to be a potential host, female beetles begin tree invasion and begin releasing aggregation pheromones, attracting hundreds of conspecifics. The simultaneous attack of many insects results in overcoming the defensive mechanism of the tree, which primarily comprises resin exudation to entrap attacking beetles. When successfully inside the tree, mating occurs, and the females begin construction of tunnels referred to as egg galleries, where eggs are deposited. A few days later, larvae hatch and feed through the summer, constructing larval galleries perpendicular to the egg gallery. The insects overwinter as mature larvae under the bark, resuming development in the spring and transitioning into the pupal stage. After transformation into the adult, the beetles remain in the tree until favorable air temperatures foster the emergence of these “brood adults”. A key event of the life cycle is the synchronized emergence of brood adult beetles within a few days. This is the result of the developmental timing of larvae [17,18,19,20] and fosters the synchronous mass attack of many trees in a short period of time.

Every year, particularly after warm winters, some beetles that attacked trees the previous year survive. These “parent adult” beetles emerge early in the year, prior to brood adult emergence, and attack new trees, attack partially attacked trees from the previous year [21] or extend galleries and oviposit in the same tree if fresh phloem is still available [18]. Either way, they apparently do not contribute measurably to population dynamics [10], yet there are scant data on the fate and roles of these parent adults. Because these parent adults emerge early in the year before brood adults emerge in synchrony, they may have a different ecological role. We hypothesize that these parent adults attack clusters of new trees and, by identifying suitable trees to attack, initiate the host selection process where brood adults may follow during their synchronized emergence. By helping brood adults identify suitable hosts, the energy associated with host finding could be minimized. In this study, we identified trees attacked early in the year by parent adults and identified which trees in the vicinity were attacked subsequently by brood adults.

## 2. Materials and Methods

### 2.1. Study Site and Sampling

The study was conducted in the Arapaho-Roosevelt National Forest in north-central Colorado, where MPB populations were at outbreak levels from the late 1990s to the mid-2010s across the National Forest. We examined eight stands for trees attacked by beetles. Stand size ranged from 0.4 to 0.64 hectares and stands were sampled during the summers of 2006, 2008, and 2015. Different stands were sampled each year. Stands were pure lodgepole pine except for one, which was lodgepole-dominated with a scattered ponderosa pine component. Beginning in May–June, stands were visited about once a week from late spring to early fall. All trees were examined during every visit, and those showing signs of new attacks, as indicated by the presence of pitch tubes and boring dust, were flagged and their locations recorded with a Global Positioning System (Garmin International, Inc., Olathe, KS, USA) or with distance and azimuth from georeferenced points. Diameter at breast height (1.37 m above ground) measurements were recorded for every tree to the nearest 1.27 cm.

**Figure 1:**
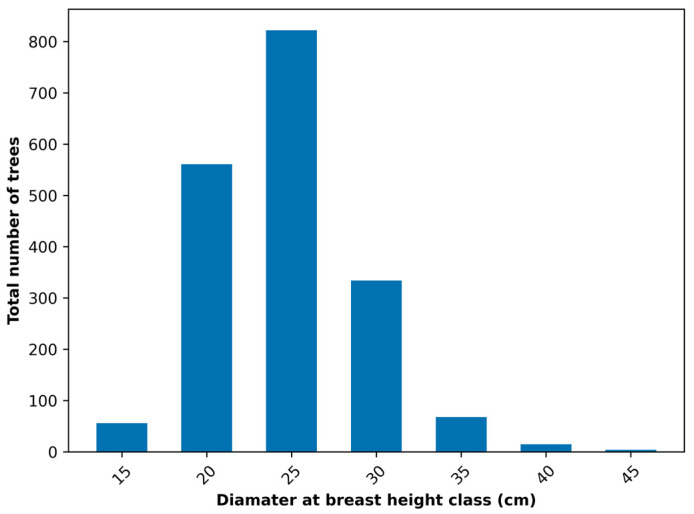
Total number of *Pinus contorta* trees attacked and killed by *Dendroctonus ponderosae* by diameter at breast height classes (cm) across all study stands, 2006–2015. Arapaho-Roosevelt National Forest, Colorado, USA.

Tree density and basal area across all stands were 466 ± 82 trees per hectare and 17.3 ± 3.0 m^2^/ha, respectively. Mean lodgepole pine tree diameter at breast height across all stands was 21.5 ± 0.6 cm. The plurality of trees attacked across all stands were in the 20 to 30 cm size classes, suitably sized trees for MPB attacks (Figure 1). All trees showing signs of attack were successfully attacked and killed (only 2 trees below the 15 cm class were attacked).

### 2.2. Data Analysis

**Figure 2:**
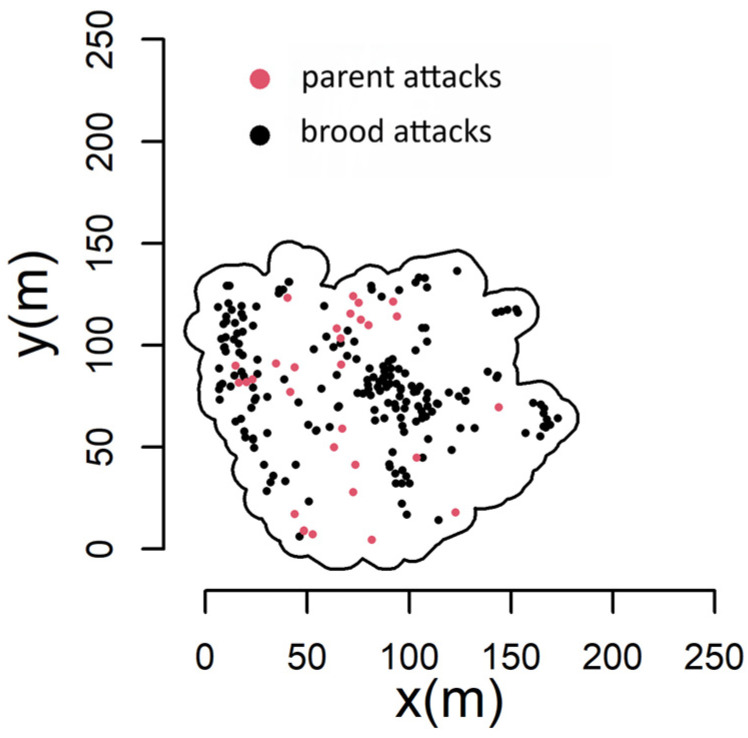
Spatial distribution of parent-attacked and brood-attacked *Pinus contorta* trees in a sampled stand. Axes represent distance (m). The concaveman R-library calculates an envelope boundary, and Ripley’s K-function is used to examine spatial patterning of attacked trees, 2006–2015, Arapaho-Roosevelt National Forest, Colorado, USA.

We tested the spatial aggregation of attacked trees. The sudden increase in the number of trees being attacked on a specific date represents the beginning of MPB brood adult emergence from trees infested the previous year. Attacks on dates prior to brood emergence are caused by parent adults that survived the winter, although some may have been early-emerging brood adults. Attacks after brood adult emergence are caused by brood adults. We examined the spatial aggregation patterns of parent-attacked trees (PATs), of brood adult-attacked trees (BATs) around PATs, and of BATs across all stands using Ripley’s K functions [22,23]. We conducted all statistical analyses in R (v4.6.0) [24]. To establish a boundary around the sampled trees, we used a concave hull algorithm using the R library concaveman [25]. The function uses a concavity argument that creates an envelope around the points to be examined to account for edge effects (https://doi.org/10.32614/CRAN.package.concaveman, accessed on 3 December 2021). A scatter plot of PATs and BATs for one of the sampled stands, along with the resulting envelope around the points, illustrates the analytical approach (Figure 2). Ripley’s K function examines the relationship between points as they may or may not change with distance, identifying if points are dispersed, aggregated, or random. Aggregation patterns are significant if the function lies above and outside of the confidence envelope for Complete Spatial Randomness, dispersed if below or random if within the acceptance band. We then fit a Thomas cluster model [26] to characterize clustering structures in terms of the number of clusters per hectare, inter-cluster distances, and cluster radius of the spatial distribution of PATs and BATs. We fit a multitype Strauss process [27] to the spatial distribution of BATs around PATs, describing their interaction in terms of inhibition.

## 3. Results

**Figure 3:**
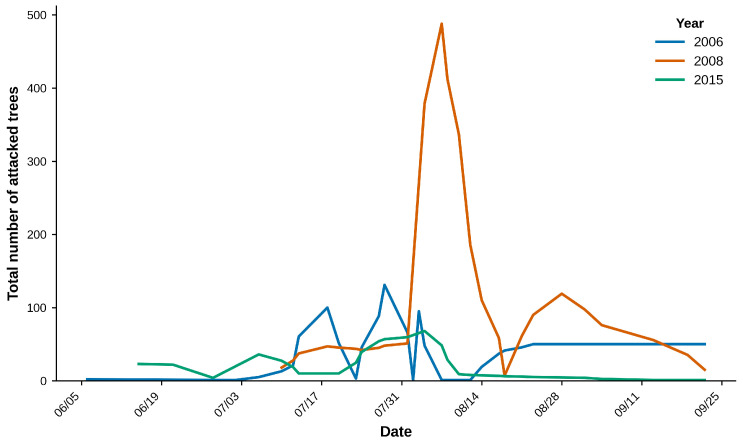
Number of *Pinus contorta* trees attacked by *Dendroctonus ponderosae* by year and sampling date (month and day), 2006–2015, Arapaho-Roosevelt National Forest, Colorado, USA.

Attacks by parent adults occurred from early June to early July. There was a notable increase in the number of attacked trees, indicative of the beginning of brood adult emergence from late July through early August. We observed attacks into September of each year. The total number of attacked trees varied over the years, with the highest numbers observed in 2008. Attack dates indicative of brood adult emergence were 18 July 2006, 7 August 2008, and 27 July 2015 (Figure 3).

**Figure 4:**
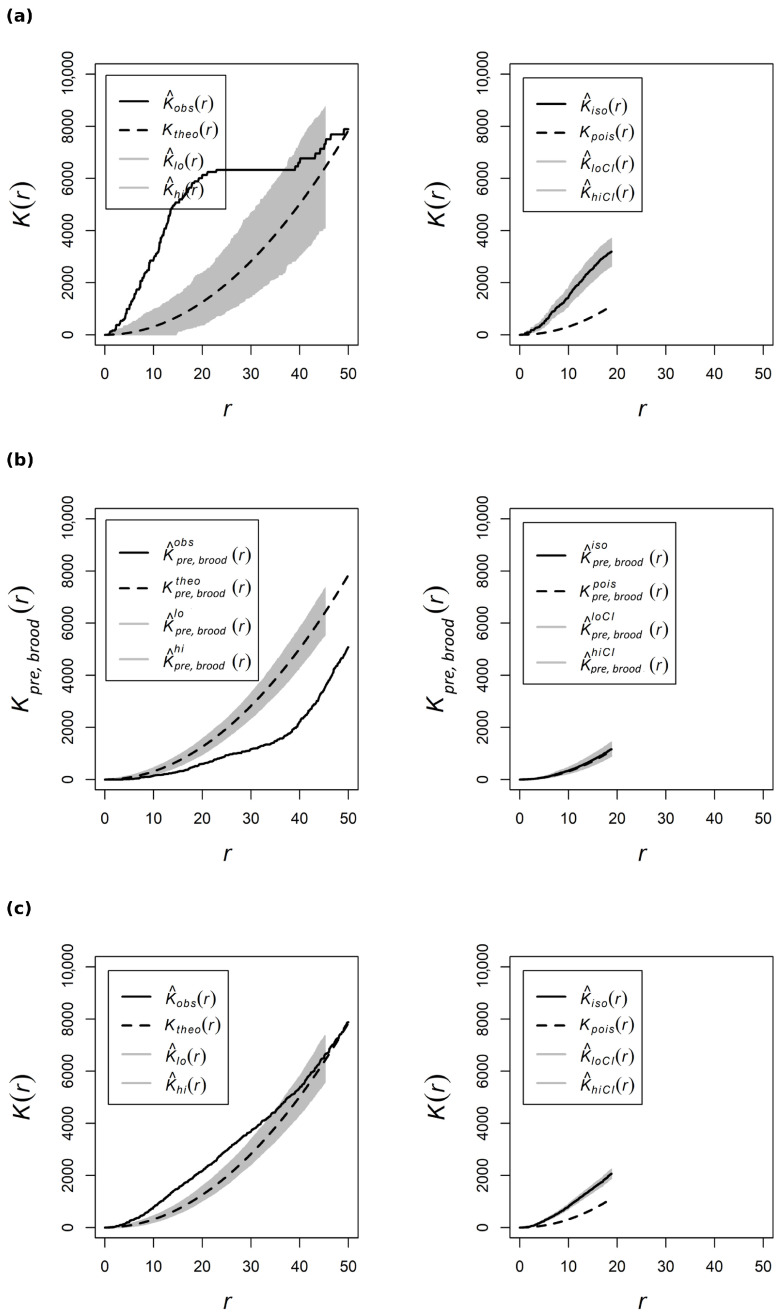
Ripley’s K-function and acceptance interval (95%) (left graphs) and bootstrap confidence bands (95%) (right graphs). X-axis (r) is distance in meters. Panel (**a**) parent-attacked trees; panel (**b**) brood-attacked trees around parent-attacked trees; panel (**c**) brood adult-attacked trees. For K-Function and acceptance interval, the solid line (${\hat{K}}_{obs}$) is the observed K-function; the dashed line (${\hat{K}}_{theo}$) is the theoretical K-function under Complete Spatial Randomness; shading represents the lower (${\hat{K}}_{lo}$) and upper (${\hat{K}}_{hi}$) 95% simulation envelope for hypothesis testing. For bootstrap confidence bands, the solid line (${\hat{K}}_{iso}$) is the estimate of the K-Function, the dashed line (${\hat{K}}_{pos}$) is the theoretical K-function (shown for reference), and shading represents the lower (${\hat{K}}_{lo}CI$) and upper (${\hat{K}}_{hi}CI$) 95% envelope for the K-function estimate uncertainty. Data from 2006–2015, Arapaho-Roosevelt National Forest, Colorado, USA.

The univariate K-function for PATs indicated strong, statistically significant clustering across all observed spatial scales (r = 5–50 m), with ${\hat{K}}_{obs}$ exceeding the upper bound of the 95% acceptance interval beginning at approximately r ≈ 5 m and remaining above it across the full range (Figure 4a) [22]. The magnitude of departure was substantial, with ${\hat{K}}_{obs}$ considerably exceeding ${\hat{K}}_{theo}$ at mid-range to large distances. ${\hat{K}}_{obs}$ appeared to plateau while ${\hat{K}}_{theo}$ continued rising, suggesting that the clustering signal persists from fine to broad spatial scales. The staircase pattern of ${\hat{K}}_{obs}$ is consistent with discrete, spatially compact cluster events rather than continuous diffuse aggregation.

The clustering structure examined by the Thomas process identified 3.64 independent PAT foci per hectare with a mean inter-cluster spacing of 56.0 m [26]. Attack foci had a mean spatial radius of 24.0 m and contained on average 16.6 attacked trees, with cluster sizes ranging from 8 to 25 trees. The estimate of the K-function falls inside the bootstrap 95% confidence interval indicating that the K-function estimate is precise across the observable range.

The cross-type K-function indicated statistically significant inhibition of BATs from PATs across virtually the entire observed range, with ${\hat{K}}_{obs}$ falling below ${\hat{K}}_{theo}$ from small spatial scales onward and dropping below the lower bound of the 95% acceptance interval beginning at approximately r ≈ 10–15 m (Figure 4b). The inhibition signal was consistent and sustained, with the departure from the acceptance interval growing progressively with r—indicating that the spatial inhibition of BATs from PATs intensifies with distance rather than operating only at a specific scale. Once again, the estimate of the K-function falls inside the bootstrap 95% confidence interval indicating that the K-function estimate is precise across the observable range.

The cross-type inhibition was characterized by a fitted homogeneous multitype Strauss process [27]. The profile pseudolikelihood identified a mean optimal interaction range of 19.5 m across all study areas, consistent with the K-function inhibition onset at r ≈ 10–15 m. The cross-type interaction parameter was estimated at γ = 0.992, representing a pairwise reduction in BAT conditional intensity of 0.82% within 19.5 m of a PAT. Although the pairwise inhibition parameter was modest, its cumulative effect across the mean PAT cluster size of 16.6 trees produces approximately a 40% reduction in BAT conditional intensity at the center of a typical PAT cluster, consistent with the sustained, broad-scale inhibition signal observed in the cross-type K-function.

The univariate K-function for BATs indicated statistically significant clustering beginning at small spatial scales (r ≈ 5 m) and persisting through 30–35 m while at larger distances the K-function falls within the acceptance band indicating CSR (Figure 4c). The magnitude of departure was smaller than for PATs and at finer scales, representing moderate clustering at fine scales but not so at broad spatial extents. The estimate of the K-function falls inside a tight bootstrap 95% confidence interval also indicating that the K-function estimate is precise across the observable range.

The clustering structure was characterized by a fitted Thomas process identifying 9.79 independent BAT foci per hectare—nearly three times the density of PAT foci—with a mean inter-cluster spacing of 33.1 m. Attack foci had a mean spatial radius of 13.4 m and contained on average 10.3 attacked trees, with cluster sizes ranging from 4 to 17 trees. Relative to PAT clusters, BAT clusters were 44% smaller in spatial extent, 38% smaller in mean size, and nearly three times more numerous per unit area. This is likely the result of dispersal of abundant brood adults occurring during elevated outbreak populations and decimating suitable hosts in the study areas.

The three independently fitted models produce a strictly ordered hierarchy of characteristic spatial scales. From smallest to largest, these were brood cluster radius (13.4 m), Strauss inhibition range (19.5 m), parent cluster radius (24.0 m), brood inter-cluster spacing (33.1 m), and parent inter-cluster spacing (56.0 m).

The brood cluster radius of 13.4 m falls entirely within the Strauss inhibition range of 19.5 m, indicating that brood clusters are spatially contained within the inhibition zone of parent attack foci. The inhibition range of 19.5 m is itself contained within the parent cluster radius of 24.0 m, indicating that inhibition is concentrated within the core of parent attack clusters rather than extending beyond their spatial footprint. The brood inter-cluster spacing of 33.1 m is of the same order as twice the inhibition range (2 × 19.5 m = 39.0 m), suggesting that brood clusters center at distances broadly consistent with displacement just beyond the parent inhibition zone.

## 4. Discussion

MPB populations were widespread and elevated during the study, and the stands sampled were suitable for infestations as they contained large-diameter trees growing in dense stands—conditions preferred by the MPB. In Colorado, lodgepole pine stands with a mean lodgepole pine dbh > 18.3 cm and basal area > 13.6 m^2^/ha have a higher probability of attack by MPBs [28,29]. Our observation of MPB attacks by parent adults early in the year and prior to the emergence of brood adults is consistent with previous studies [18,21,30].

We observed spatial aggregation of PATs, which has not been previously reported empirically. Spot proliferation, meaning the initiation of new infestations away from a current infestation, occurs most frequently at distances of 30 m and 50 m [31]. The observed inter-clustering distance of 56 m for PATs is consistent with these reported distances, suggesting the occurrence of spot proliferation by parent adult beetles and that these beetles can disperse and are not restricted to adjacent trees. Yet, we also observed clustering at shorter distances than 30 m for PATs. These shorter distances can represent spot growth of infestations, when beetles attack new trees in the same area. MPBs utilize stored reserves of lipids, proteins, and carbohydrates for dispersal flights [32]. Shorter distances may be the result of reduced energy reserves of lipids [30] or simply the availability of susceptible hosts in proximity. Reduced energy reserves of lipids in parent overwintering adults have also been demonstrated for the spruce beetle, *Dendroctonus rufipennis* [33], the southern pine beetle, *Dendroctonus frontalis* [34], and the European spruce bark beetle, *Ips typographus* [35].

Aggregation of PATs also suggests that surviving beetles from overwintering can complete successful host selection and produce aggregation pheromones, resulting in clusters of attacked trees. During the initial stages of host selection and colonization, MPBs exhibit random landings on the surface of the bark [36], responding to visual stimuli from vertical objects [37] and to gustatory stimuli [38]. The beetles also detect secondary monoterpenes emitted by host trees [39]. MPB attacks on host trees are initiated by the females and are regulated by a complex chemical communication process [16,40,41]. When females excavate through the bark to gain access to the phloem, they consume α-pinene, which is metabolized through oxidation into the pheromone *trans*-verbenol, which is attractive to males. The males then produce and release *exo*-brevicomin, which is attractive to females. Myrcene and terpinolene, host monoterpenes, augment the attraction, resulting in mass attacks; many attacking beetles contribute to depleting resin resources, the primary defensive mechanism of the tree. Our findings suggest that parent adults can complete this process, except for the mass attack, as we did not observe these by parent adults. There were likely not enough parent adults. Regardless, parent adults lack the ability to produce offspring, not contributing to population dynamics [42].

There was no aggregation but rather an inhibition of BATs around PATs, rejecting our null hypothesis. Brood adults did not use trees attacked by parent adults as a guide for the initiation of new groups of attacked trees. This may be due to brood adults not responding to pheromones produced by parent adults. Beetles need a flight period before they become responsive to pheromones. In addition, pheromones produced by parent adults may be at low levels as the beetle population is low prior to brood adult emergence, contributing to a muted response [10,16,30].

We observed clustering of BATs at similar fine scale distances to PATs indicative of growth of existing infestations. As for BATs, CSR at distances of 30–50 m are representative of spot proliferation while short distances are a function of the availability of suitable hosts close to previously infested hosts [43,44] and are the result of beetles being redirected to adjacent trees by anti-aggregation pheromones after a tree becomes fully colonized [16,40,41].

The spatial analysis of PATs, BATs around PATs, and BATs reveals a coherent and spatially structured dynamic of attacks operating at the stand scale. PATs and BATs form discrete clusters because of the host colonization process. BAT clusters were more numerous, associated with the availability of susceptible hosts. BATs are inhibited from parent attack centers. The convergence of independently estimated spatial scales across all three processes supports a spatially structured dispersal and re-aggregation of dispersing beetles.

An important finding of this study is the clustering of PATs. Researchers conducting future studies of MPB dispersal or of the spatial distribution of attacks need to be cognizant of the spatial patterning of attacked trees to make sure the correct target population is being sampled. Biological features of MPB and other bark beetles warrant re-examination, as our previous understanding of the ecology of the insects may evolve under novel climatic conditions. In general, insect responses to climate change are anticipated to be positive, such as expanding geographical distributions, faster generations, and increased fecundity, yet negative responses, such as phenological mismatches with their hosts or reduced nutritional quality, and unknown interactions with associates could occur [45,46,47,48]. The MPB is already exhibiting range expansion associated with increased temperatures [49,50,51,52,53] and colonization of novel hosts [54]. Therefore, life history characteristics of mountain pine beetles need to be viewed under the umbrella of climate change.

## Data Availability

Data in support of this study will be available upon manuscript publication at the USDA Forest Service, Research Data Archive (https://www.fs.usda.gov/rds/archive/) by searching for the title of the article.
